# History of Several Cases of Phthisis Pulmonalis Treated with Mercury

**Published:** 1807-10

**Authors:** W. Watson


### *History of several Cases of Phthisis Pulmonalis treated with Mercury, by* Mr. W. Watson; *in a Letter to* Dr. Coxe, *of Philadelphia.*

THE three following cases of phthisis pulmonalis were treated with mercury. In one case the mercury was ineffectual; but this case can furnish no objection to its use; on the contrary, it irresistibly enforces more seasonable application. Although the treatment of pulmonary consumption by mercury is pretty generally known, yet there still prevails such a degree of scepticism of its efficacy, amongst many of the physicians distant from the city, as to render the publication of cases, as they occur, highly necessary. Some have used it without success, which has deterred them from a second attempt. But it is not to be supposed that it can effect a cure in every instance. Other diseases, of much less force, have destroyed life, notwithstanding they have had their appropriate remedies. It is to be hoped that the doubts of medical men on this subject will shortly be dissipated; and to furnish my mite, the subsequent cases are submitted.

I was called, on the 8th of February, 1804, to visit E. W. Hale, Esq. counsellor at law, a young gentleman about twenty-seven years of age, who, a few days before, had been attacked with pneumonia from great exposure to cold. He was able to walk about, but, contrary to his usual appearance, was very much dejected; complained of lassitude, fever, and hard and dry cough, but unaccompanied panied with pain in any part of the thorax; the pulse small but tense. I conceived his disease to be a bad catarrh, but having suspected for some months previous that his lungs were in a very weak state, I felt a good deal apprehensive of the event.

He was blooded, and took an emetic of ipecacuanha. His blood exhibited very evident marks of inflammation, and his pulse rose after the operation. I left him a few pills, composed of digitalis and opium, from which, in former cases of this disease, I had experienced very happy effects; and as I lived twelve miles distant, I directed him to be blooded next morning, if the cough and stricture were not relieved. He was blooded the next morning, and I visited him on the 10th. He had found no relief from the remedies mentioned, and the indication for blood-letting was greater than at any time before. I bled him; the blood was sizy and coated, as in pleurisy. I left him some antimonial powders, compounded of nitre, tartarized antimony, and calomel, in the proportion directed by Dr. Rush. He was to take one every second hour till my return, and also to be blooded every day, if fever and stricture were not abated. On the 12th I visited him; he seemed much better; was able to walk about; but as the cough was still hard and dry, with stricture, he was again blooded, and directed to have the operation performed daily, till he could expectorate with freedom. The powders were repeated, so as to purge and sweat him. On the 17th he sent for me. I found him much worse; he was unable to leave his bed, fever very high, his cheeks efflorescent, and his pulse frequent and tense. I bled him, and remained the night with him, and the next morning bled him again. The blood was very inflammatory, but there was still no pain of the chest. The antimonial powders were continued; they sweated and purged him, but neither the cough, fever, nor stricture, were relieved. The 20th I saw him; he complained much of weakness and a distressing tickling cough. His pulse still indicated blood letting; the blood very inflammatory. I gave him a mixture of nitre and spermaceti in the yolk of an egg, which appeased the tickling. The arterial action was now less convulsive, fever much moderated; but the cough and oppression of the præcordia continued, with much distress. From the commencement of the disease, he had expectorated after coughing, a little thin acrid matter mixed with striæ of florid blood. I now applied a blistering plaster over each lobe of the lungs, so large as to cover all the anterior anterior portion of the thorax. While the blisters were rising, he sweated most profusely. The blisters rose well, and excited severe strangury, which I did not attempt to relieve as long as it could be borne, having observed, in fevers, the happiest effects from this symptom, in determining excitement from parts more essential to life. The strangury was removed by the spiritus nitri dulcis. The cough was now very distressing, and expectoration by no means free; but there was a total change in the action of the pulse. It was faltering and thread-like, and the coughing harassed him so much, that I was apprehensive he could not survive many hours. Profuse sweating and frequent deliquia animi: laudanum was prescribed, and to be repeated after short intervals, to prevent the torpor of indirect debility. He took a little Madeira wine frequently, and sago gruel for food; with these he was supported for several days. From his cough, hoarseness, stricture, and the matter expectorated being streaked with blood, I was satisfied the disease had degenerated into phthisis pulmonalis. At this time he was as much emaciated as I had ever known a patient to be in the typhus state of the same disease.

It was then twenty-two days from the period of attack; I had no hopes of a recovery. He was unable to change his position in bed. A severe hectic visited him every afternoon, and he could not speak to be understood. He took wine, laudanum, opium, and sometimes bark; but they afforded a very temporary relief.

I had seen, some time ago, in the Medical Repository, three letters from Dr. Rush to Dr. Miller, on the efficacy of mercury and tonic remedies in pulmonary consumption. I had attempted this medicine with others, but they were too much exhausted before they would submit to its administration. I now began it with Mr. Hale, but doubted much whether ptyalism could be excited. I dreaded that the excitability was too far exhausted to favour the operation of any remedy.

Half an ounce of strong mercurial ointment was directed to be rubbed into his sides and breast every night, and two grains of calomel, with half a grain of opium to be taken every fourth hour. This prescription was attended to till the 26th, when I saw him: the mercury had produced no affection of the stomach or intestines. He had become costive. In addition to former directions, another half ounce of the ointment was to be rubbed into the same parts every morning. The 28th, he had still no dejection jection from the bowels; two laxative enemata were administered in the course of the evening, which removed the constipation. The mercury was continued without any evident effect, either upon his mouth or upon his breath. In addition to the mercury already prescribed, four grains of calomel were directed to be taken morning and evening, mixed with a little honey, which was to be dissolved in the mouth, with the intention of exciting action in the gums and salivary glands. On the 4th, there were no symptoms of mercurial disease; the medicine did not even purge him.

The mercury had now been taken for eleven days, in the quantities mentioned. He ceased to take it internally, but two ounces of mercurial ointment were directed to be rubbed into his sides, breast, and thighs, every twenty-four hours, and Madeira wine and laudanum to be given in such quantities, and repeated after such intervals, as might support the continued excitement of the medicine. They were prescribed with the intention of raising the system to that point, which would favour the salutary operation of mercury. 6th, No symptoms of ptyalism. 9th, The same; cough distressing, copious expectoration of pus and florid blood, excited with deep, sonorous, and difficult coughing. Hectic fever very high every afternoon.

The vascular system, as well as the muscles, were in the most extreme state of debility. The mercury was still continued externally, and bark given to support a more durable excitement, and laudanum to appease the distress of coughing. His common drink, during the whole course of this treatment, was malt beer, in the usual strength. 11th, No change of situation. Bark did not agree well with his stomach, and wine, soups, and opium, were administered as a substitute. 14th, I visited him,—the mercury had not acted.

I then consulted, by letter, my ingenious friend Dr. Harris, of Bellefont, who, I had heard, had been successful in the cure of Phthisis Pulmonalis, with the mercurial treatment. He advised the use of more durable stimulants, as columbo, gentian, &c. and the application of flannel bandages saturated with mercurial ointment, to the breast and sides, after removing the skin by blisters; but to precede the tonics with a nauseating dose of tartarized antimony, to excite new action of the stomach. A trial of the carbonic acid gas, as directed by Dr. Beddoes, was also recommended, either as a means of depriving the lungs of the usual quantity of oxygen, the principle of inflammation and ulceration, or as a vulnerary. The nauseating dose was immediately prescribed, the blister applied to the breast and ribs, and columbo given in substance, mixed with beer. This was done on the 17th. 19th, His condition as usual. The saturated bandages were now applied. Calomel was rubbed into the mouth and gums, and mercurial ointment was rubbed into his throat. He inhaled, during several days, the carbonic acid gas from a tube placed in his mouth, when it was discontinued, as well from its apparent inutility, as from a supposition that it might impede the ptyalitic excitement of mercury by disoxygenating the system. He alternated the durable stimulants, columbo, gentian, orange peel and bark, with one another, till the 26th, when calomel and opium were given in doses of a grain each, every fourth hour. The stimulants were continued, the calomel rubbed into his cheeks and gums twice a day, and the ointment into the throat as often. The bandages were renewed after being saturated, every twenty-four hours.

His mouth had been sore from the commencement of the disease; but it now exhibited evident symptoms of the operation of mercury; his breath also indicated the action of the medicine; but all the symptoms of pulmonic disease continued with unabated violence. 29th, His mouth ulcerated, but no appearance of ptyalism. April 2nd, The salivation had not begun, nor did his mouth appear more affected. From the 21st ultimo I had discovered soreness of the gums from the mercury. At this time, I had it in view to try the nitric acid, to hasten the ptyalitic action, but when I returned on the 4th, the salivation had begun.

Thus, after having spent *forty-two* days in the most assiduous attempts to salivate him, with unspeakable joy I succeeded, after having prescribed two hundred and twenty-two grains of calomel, and thirty ounces of mercurial ointment, composed of two parts axunge, and one part hydrargyrus. The stimulants mentioned were continued, and the mercury as before, and he had used a cordial and stimulating diet, from the transition of excessive to deficient excitement of the muscular and vascular systems.

8th, He salivated about two pints in the twenty-four hours. The cough had abated in frequency and violence. The saturated bandages were suffered to remain, but the calomel and opium were withheld. 12th, The salivation had increased to three pints, the cough perfectly easy, and the the quantity of pus expectorated, much diminished. 16th, The mouth and fauces very much swelled, salivation very profuse. The columbo, &c. were withheld, and laudanum directed to be frequently taken, and a gargle of borax and honey dissolved in tepid water, diligently used. 19th, The discharge of purulent matter had disappeared, and the cough had entirely left him. At this time, from two to three quarts of saliva daily flowed from his mouth. The emaciation was extreme, and he had not as yet been able to raise himself from his bed. His appetite good.—He now took columbo in infusion, cordial diet, and laudanum. The saturated bandages were directed to be removed; but owing to a mistake in the attendants, they were suffered to remain on till the 1st of May. At this time he could walk through the room, although the salivation had not abated in the least, and the emaciation was, if possible, greater. The bandages were now removed. He continued the columbo, gentian, &c. and on the 11th, the ptyalism began to abate, but did not altogether leave him till the 15th of June—all which time he took the durable stimulants in diminished quantities, cordial diet, wine, beer, and London porter.

The recovery of health was rapid; but hoarseness and weakness of the voice remained. He was directed to take garlic daily, which removed these symptoms about two months after. He is now more lusty than he had ever been before; but occasionally feels a pain in his breast from close reading or writing, which leaves him when he rides or exercises. From the 22nd of February he drank half a gallon of beer daily. Five weeks of the time he was under the operation of mercury, he never spoke, on account of the tumefaction and soreness of his mouth, tongue, and fauces.

He had the remittent fever slightly, the autumn before he was attacked with pneumonia; till that time he had been very healthy, and somewhat corpulent. The disease was not hereditary, nor had he any appearance of mal-formation of the thorax. Since his recovery, he has been perfectly healthy, except that he had a few fits of vernal intermittent, in the last month.

I was called on the 10th of April, 1804, to visit Mrs. Irvine, a married lady of delicate habit, about thirty years of age, who had been afflicted with cough and pain of the chest for eighteen months before. She was confined to bed, much emaciated, with incessant cough, and purulent expectoration; no appetite, hectic fever in the afternoon, afternoon, pulse quick, frequent and irregular, oppression and dyspnœa, especially on the slightest motion. She had borne three children, and from the birth of the last, she had dated her disease.

I took away a few ounces of blood, gave her the antimonial powders, and directed two drachms of mercurial ointment to be rubbed into her sides and breast every night. 13th, Her mouth sore, gums swelled, and ptyalism begun. I was suddenly called to her to-day, when I found her in an hysteric paroxysm. I gave her a few drops of vitriolic æther, which, after two or three repetitions, relieved the paroxysm.

About two o'clock the next morning, I was sent for, in the utmost haste, to visit her. When I arrived, I found the attendants supporting her in the bed, in a fainting fit. In her sleep she had been attacked with menorrhagia, and the discharge was so profuse as to induce deliquium, before it was discovered. No pulse could be distinguished, and the discharge continued without abatement. I gave her a little laudanum, and directed one of the attendants to introduce flour into the vagina, and press upon it with the hand; and as soon as she could swallow with freedom, I gave her kino and alum rup. She took them in such quantities, and repeated with as much frequency as her stomach would bear. The discharge, however; did not abate. I was afraid to apply cold water, by reason of the mercury; but I soon saw it must be used or my patient must sink. Cold water was then applied to the abdomen, perinæum, &c. the windows were opened, and she was directed to drink cold water. The disease abated soon after, and ceased in two or three days. I looked forward with much anxiety for the consequence; but on the 15th her throat, fauces, tongue, &c. swelled astonishingly, but her breathing was not much impeded, and I was now relieved from my apprehensions. The tumefaction of the throat was so great as to prevent swallowing. For ten days she was supported by injections of broth; but the swelling then gave way to blistering on the back of the neck and around the throat, and borax as a gargle. 22nd, Salivated about a pint daily: the mercurial frictions were continued. May 1st, Salivation profuse, cough and purulent expectoration much abated. 9th, Cough and expectoration ceased. 14th, Mercurial frictions were discontinued, and an infusion of columbo with wine and a cordial diet, were recommended to be taken. She was excessively weak, but after this time recovered strength daily. 26th, Salivation

Salivation abated, which left her the 7th of June. She continued to take columbo, gentian, bark, and orange peel, in succession, till the 1st of August, when her strength was quite restored. She is now much more healthy and strong than she has been for several years.

Miss Lyon, a young lady of twenty three years of age, put herself under my care in May, 1804. She laboured under phthisis pulmonalis for eighteen months or two years. She was much emaciated, had hectic fever, night sweats, did not cough much, nor did her cough agitate her; but she expectorated very freely, had great weakness, and was very averse from motion or exercise. I gave her calomel and opium, and directed mercurial ointment to be rubbed into her sides and breast. The mercury produced no effect. A large blister was applied to her breast and ribs, and saturated bandages applied as in the case of Mr. Hale, but still without effect. Blisters were then applied to the inner sides of her thighs and under her arms, and dressed with mercurial ointment and calomel united. She took infusion of columbo through the day, in such quantities as her stomach would bear, and opium at night. These remedies were continued for six weeks without any abatement of pulmonary disease, and without exciting the least action in the gums, fauces, throat, or salivary glands, when she died a lamentable instance of too late application;—the excitability being exhausted below that point, in which mercury will excite disease.

